# Scalable Solvent-Mediated Nanoarchitectonics of High-Surface-Area Mesoporous Ni_2_P_2_O_7_ for Enhanced Electrochemical Performance in Alkaline Media

**DOI:** 10.1021/acs.inorgchem.5c05373

**Published:** 2025-12-26

**Authors:** Gözde Ceran, Irmak Karakaya Durukan, Işıl Ulu, Ömer Dag

**Affiliations:** † Department of Chemistry, 52948 Bilkent University, Ankara 06800, Turkey; ‡ UNAM—National Nanotechnology Research Center and Institute of Materials Science and Nanotechnology, Bilkent University, Ankara 06800, Turkey

## Abstract

Sol–gel synthesis provides a versatile and scalable route for producing high-surface-area materials. Here, we develop a facile sol–gel strategy for mesoporous nickel pyrophosphate (Ni_2_P_2_O_7_) using H_4_P_2_O_7_, nickel nitrate, and Pluronic P123 in butanol- and ethanol-based media. These mixtures form homogeneous sols that gelate and, after drying and calcination at 300 °C, yield powders with surface areas up to 410 m^2^ g^–1^. In contrast, methanol systems—and certain ethanol conditions—phase-separate into precipitate and supernatant layers. After calcination, the solution and precipitate fractions produce mesoporous Ni_2_P_2_O_7_, while the supernatant fraction forms Ni_3_(PO_4_)_2_ with different textural properties. The materials remain amorphous up to 600 °C and crystallize into α-Ni_2_P_2_O_7_ at 700 °C. Thin-film electrodes were prepared by spin-coating on fluorine-doped tin oxide and dip-coating on graphite, followed by calcination. In 1 M KOH, Ni_2_P_2_O_7_ converts into ultrafine Ni­(OH)_2_ nanoflakes, with the transformed graphite-supported electrodes showing high oxygen evolution reaction (OER) activity and stability. Moreover, Ni_2_P_2_O_7_ is stable in alkaline media (pH ∼13) when the P_2_O_7_^4–^ concentration exceeds ∼0.27 M in the electrolyte. This work demonstrates a simple, tunable route to mesoporous Ni_2_P_2_O_7_ and reveals its conversion into highly active OER electrocatalysts (381 mV at 100 mA cm^–2^, 45 mV dec^–1^).

## Graphical abstract

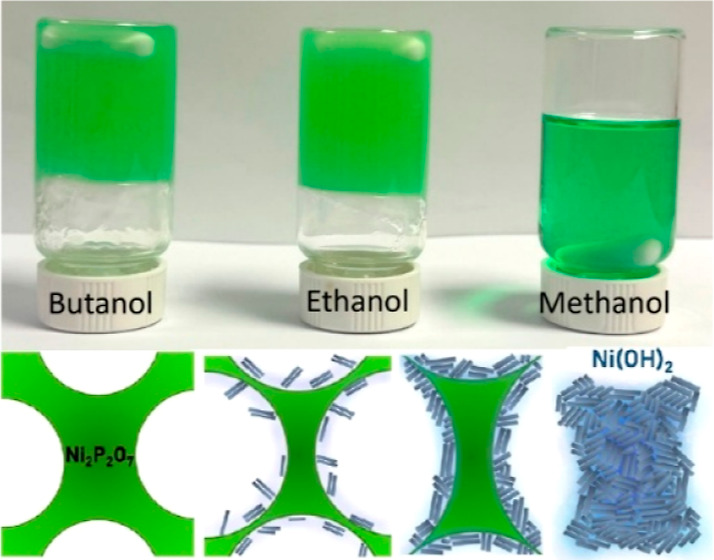

## Introduction

Nanoarchitectonics is an important concept in the design and structuring of materials at the nanoscale to practical length scales.^1–3^ Structural hierarchy in material design, with control at every length scale—from molecules to clusters, to nanoparticles, and further to nanoscale and microscale assemblies—requires chemical processes capable of creating technologically important yet synthetically challenging materials. The technology behind such assemblies can be described as nanoarchitectonics.^1–3^ Nanoarchitectonic mesoporous materials can be designed by carefully controlling the underlying principles of this field and are highly valuable in applications that require large surface areas.^4–8^ Among these, transition-metal phosphate (MP) pyrophosphates (MPPs) and metal oxides/metal hydroxides are particularly important and have been extensively investigated for applications in electrocatalysis, battery technologies, and supercapacitors.^8–20^ However, there are only a limited number of methods available for synthesizing mesoporous MPs and MPPs with high-surface areas^21–24^ (see Scheme 1).

**Scheme 1:**
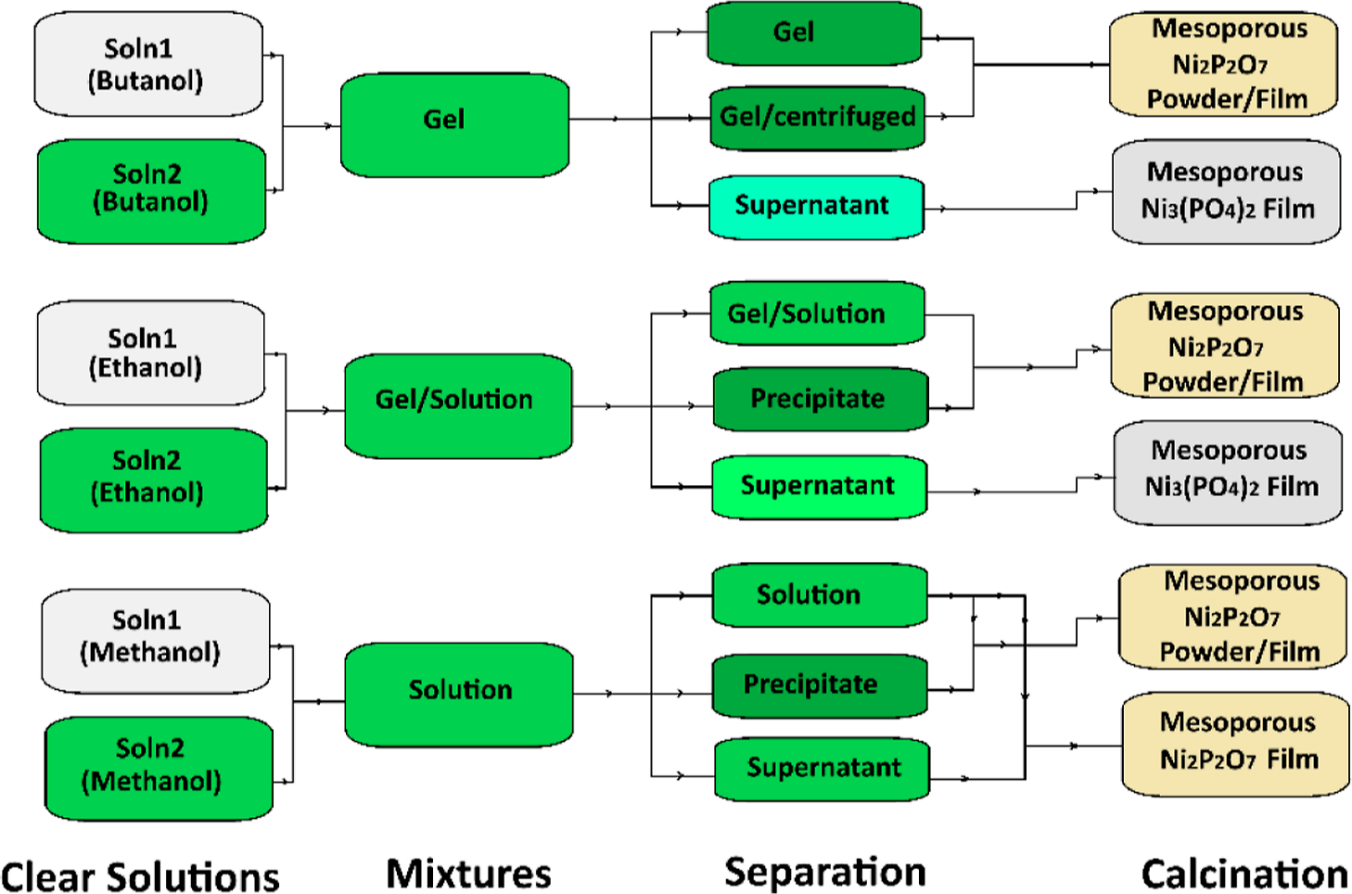
Schematic Representation of the Synthesis Protocol in All Three Solvent Systems (Butanol, Ethanol, and Methanol)^a^

a Dark green and light green represent high and low Ni­(II) concentrations.

One of the main challenges in achieving ordered mesoporous structures is the difficulty in simultaneously controlling the particle formation and assembly kinetics of MPs and MPPs.^25–29^ Transition-metal ions react rapidly with phosphate or pyrophosphate ions, leading to fast precipitation and particle growth, which occurs too quickly for surfactant molecules to direct the assembly into mesostructured frameworks. Consequently, currently available soft-templating approaches have largely failed to yield MP and MPP materials with a controlled mesostructure and stable mesoporosity. Several studies have attempted to mitigate this issue by modifying the sources of metal and phosphate ions.^25–30^

Recently, we demonstrated a soft-templating approach in which phosphoric acid (H_3_PO_4_, PA) and pyrophosphoric acid (PPA) (H_4_P_2_O_7_, PPA) can serve as effective sources of phosphate and pyrophosphate, respectively.^31–33^ For example, aqueous solutions containing PA (or PPA), metal salts, and HO­(CH_2_CH_2_O)_20_-(CH_2_CH­(CH_3_)­O)_70_-(CH_2_CH_2_O)_20_H, Pluronic P123, surfactant can form semistable lyotropic liquid crystalline mesophases. These mesophases can be coated onto various substrates, slowly transforming into mesostructured M_2_H_*x*_(P_2_O_7_) (NO_3_)_*x*_·nH_2_O soft or semisolid materials, which can then be calcined to produce mesoporous MPs or MPPs—albeit with relatively low-surface areas.^32,33^ Reported surface areas from such methods typically range between 35 and 110 m^2^/g.^31–33^

The relatively low-surface areas are attributed to the rapid growth of MP or MPP particles, which prevents proper assembly by the surfactant species, resulting in thicker pore walls. However, if the particle size of the growing MP or MPP can be precisely controlled, then the pore wall thickness can potentially be reduced to just a few unit cells. For instance, a material with wall thickness limited to a single unit cell could theoretically achieve surface areas exceeding 6000 m^2^/g. That said, such extreme porosity may result in surfaces terminated by hydroxide or P–OH groups, which might alter the material’s functionality compared to typical MPPs.

Therefore, new strategies are needed to control the pore wall thickness—either by slowing the growth kinetics of MPP particles or by enhancing the assembly process (e.g., increasing the micelle concentration in the medium). Achieving this will require the development of new precursors (both metal salts and pyrophosphate sources) and carefully designed synthesis environments (choice of solvent, pH control through acidic or basic media, etc.). These parameters need to be investigated toward producing large-surface area mesoporous MP and MPPs.

Among mesoporous MPPs, mesoporous Ni_2_P_2_O_7_ stands out for its exceptional performance in electrocatalysis and supercapacitors.^8,9,13,32,33^ It exhibits outstanding oxygen evolution reaction (OER) activity and high charge capacity in alkaline media. However, Ni_2_P_2_O_7_ transforms into Ni­(OH)_2_ under alkaline conditions.^33^ The remarkable electrocatalytic performance and high capacity are believed to result from the formation of ultrasmall Ni­(OH)_2_ particles.^32,33^ Thus, synthesizing highly porous Ni_2_P_2_O_7_ may allow for control over this transformation process. Mesoporous MPPs may also serve as precursors for producing metal hydroxides with tailored size and morphology. In particular, nickel hydroxide exhibits high charge storage capacity and excellent electrocatalytic activity for the oxidation of various compounds.^34–39^

In this study, we demonstrate that alcohols are unique and effective solvents for the sol–gel synthesis of mesoporous Ni_2_P_2_O_7_. We tested methanol, ethanol, and butanol and found that these alcohols play a crucial role in controlling the self-assembly process. When a nickel salt solution (metal salt, Puluronic P123, and alcohol) is mixed with an acid solution (PPA, Puluronic P123, and alcohol), a clear homogeneous solution forms, which rapidly transforms into a gel. Upon calcination, this gel yields high-surface-area mesoporous Ni_2_P_2_O_7_ particles—with a surface area of up to 410 m^2^/g.

## Experimental Methods

### Materials

Pyrophosphoric acid (PPA, ≥94%), nickel­(II) nitrate hexahydrate ([Ni­(H_2_O)_6_]­(NO_3_)_2_, 99.9%), poly­(ethylene glycol)-*block*-poly­(-propylene glycol)-*block*-poly­(ethylene glycol) (EO_20_-PO_70_-EO_20_, Pluronic P123, *M*_w_ ∼5800 g mol^–1^, 99.9%), fluorine-doped tin oxide (FTO) glasses, and graphite rods were purchased from Sigma-Aldrich, and the solvents (absolute ethanol (99.9%), methanol (99.9%), and 1-butanol (≥99.0%)) were purchased from ISOLAB. All chemicals were used without further purification.

### Preparation of Ni­(II)/PPA/P123 Solutions

The sol–gel solutions were prepared by dissolving [Ni­(H_2_O)_6_]­(NO_3_)_2_/PPA/P123, 30:15:1 mol ratio in 10 mL of the chosen solvent. In a typical solution preparation, after 1.504 g of nickel nitrate salt is first dissolved in 5 mL of alcohol, a 0.5 g of the surfactant is added to this solution and stirred until a clear green solution is obtained. In another vial, 0.406 g PPA is dissolved, then a 0.5 g of the surfactant is added and stirred to obtain a clear colorless solution. Having homogeneous solutions in both of these vials, the acid solution is slowly added to the nickel salt solution. After shaking the solution very gently for a few seconds, then the mixture is kept undisturbed and within few seconds, a stable gel formation is observed.

### Preparation of Ni­(II)/PPA/P123 Mesophases

The sol–gel solutions and supernatant parts (after centrifugation at 6000 rpm for 15 min) are coated on microscope glass slides via the drop-casting method. However, the gel-like precipitate is spread onto microscope slides by squeezing the gel in between the two microscope slides. Then, these samples were used for further characterization and treatments.

### Synthesis of Mesoporous Nickel Pyrophosphates

The sol–gel solutions are directly poured onto pyrex dishes and dried at 100 °C for 24 h. After drying, the powder is first ground in a mortar and then transferred to a combustion crucible and calcined at higher temperatures (300, 400, 500, 600, and 700 °C) for 1 h. The supernatant part is drop-cast coated on microscope glass slides and then calcined at 300 °C. The calcined samples were collected by scraping from glass slides as powders. Then, the collected powders were further annealed at higher temperatures. The gel-like precipitate part is first dried at 100 °C for 24 h in centrifuge tubes. Then, the resulting powder is first ground in a mortar, transferred to a combustion crucible, and calcined at various temperatures (300–700 °C).

### Fabrication of the Mesoporous Ni_2_P_2_O_7_ Electrodes

The electrodes used for electrochemical characterization are either dip-coated onto a 1 cm^2^ area of a polished graphite rod (3 mm in diameter) using the above solutions and supernatants by 5-fold diluting (dip-coating was performed under ambient laboratory conditions at room temperature and ∼30% relative humidity, with a withdrawal speed of 1.45 mm s^–1^), or spin-coated onto FTO substrates at 2000 or 5000 rpm. Because the dip-coating method produces relatively thicker films on the substrate, the coating solutions were diluted 5-fold with the corresponding alcohol. After coating, both the graphite and FTO electrodes were calcined in a preheated oven at temperatures ranging from 300 to 700 °C for 1 h.

### Time-Dependent Investigation of Ni_2_P_2_O_7_ to Ni­(OH)_2_ Transformation

0.1 g of Ni_2_P_2_O_7_ powder is put in a centrifuge tube with 10 mL of 1 M KOH. The samples are removed by centrifugation at 6000 rpm immediately, 15, 45 min, and 1 day aging in 10 mL of 1 M KOH solution. Upon removal of the supernatant of the centrifuged samples, they are washed with distilled water four times to remove K^+^ ions and other impurities from the sample. Finally, each sample is dried at 80 °C for 1 day and used for characterization.

### Characterization

#### Attenuated Total Reflectance Fourier-Transform Infrared Spectra

The attenuated total reflectance Fourier-transform infrared (ATR-FTIR) spectra were recorded with a resolution of 4 cm^–1^ and 64 scans in the 400–4000 cm^–1^ range using a Bruker Alpha Platinum ATR-IR spectrometer with a Digi Tect TM DLATGS detector. The spectra were collected by placing a few drops of the solution or supernatant for the analysis of gel phases or calcined powder on the diamond ATR crystal.

#### Polarized Optical Microscopy

Polarized optical microscope (POM) images of the gel-like films were recorded using a ZEISS Axio Scope A1 polarizing optical microscope.

#### X-ray Diffraction Patterns

Small- and wide-angle X-ray diffraction (XRD) patterns are recorded using a Rigaku Miniflex diffractometer, equipped with a Cu Kα (1.54056 Å) X-ray source, operated at 30 kV and 15 mA. The small-angle XRD patterns are recorded between 1 and 5°, 2θ, with a scan rate of 0.5°/min. The wide-angle XRD patterns of the films are recorded between 10 and 80°, 2θ, with a scan rate of 0.5°/min. The wide-angle XRD patterns of powders are collected by using a Panalytical multipurpose X-ray diffractometer equipped with a Cu Kα (1.54056 Å) X-ray source, operated at 45 kV and 40 mA.

#### X-ray Photoelectron Spectroscopy

XPS spectra are recorded using a Thermo Scientific K-alpha photoelectron spectrometer equipped with an Al K_α_ monochromatic source (1486.68 eV) and a 400 μm spot size. All of the spectra are calibrated according to the C 1s peak at 284.8 eV, and elemental percentages are analyzed using the XPS survey spectra.

#### Scanning Electron Microscopy

Scanning electron microscopy (SEM) images of the films and powders are recorded using (FEI) Quanta 200 F scanning electron microscopy at 15 keV beam energy.

#### N_2_ (77.4 K) Adsorption–Desorption Isotherms

Before the measurement, the powder samples are dehydrated under vacuum conditions at 200 °C for 2 h. Then, the isotherms are collected by using TriStar 3000 (Micrometrics) in the relative pressure range of 0.01 to 0.99 atm.

#### Electrochemical Analysis

Electrochemical experiments were performed using a Gamry Instruments (Potentiostats PC14G750 and IFC5000-07565). Three electrode system (namely, Ag/AgCl (3.5 M KCl) as the reference electrode, RE, a platinum wire as a counter electrode, CE, and FTO and graphite rod-coated Ni_2_P_2_O_7_ electrodes as a working electrode, WE) was used in a 30 mL 1 M KOH solution in a polypropylene (PP) cell. The potential is converted and reported with respect to that of the normal hydrogen electrode (NHE).

Before all the electrochemical measurements, nitrogen gas is purged into the electrolyte solution for 15 min to remove any dissolved oxygen. The measurement is initiated by cyclic voltammetry (CV) to evaluate the stability of the working electrodes. Subsequently, multistep chronoamperometry (CA) is performed by sequentially increasing the potential in 0.01 V increments. Tafel slope data are extracted from the plot of log­(*j*) vs overpotential. Finally, chronopotentiometry (CP) experiments are conducted at constant current densities ranging from 1 mA/cm^2^ to 100 mA/cm^2^ to determine the overpotential values for OER at different current densities and to assess the stability of the working electrode in alkaline media.

#### Electrochemical Investigation of Ni_2_P_2_O_7_ to Ni­(OH)_2_ Transformation

The same electrodes are used to monitor the Ni_2_P_2_O_7_ to Ni­(OH)_2_ transformation using a mixture of a 15 mL of 2.4 M KOH and 15 mL of 0.6 M H_4_P_2_O_7_ electrolyte solution (neutralized solution). Five CVs are recorded in the above neutralized solution and after adding 1 M KOH with an 0.5 mL increment (0 to 15.5 mL).

## Results and Discussion

A nonionic surfactant, P123, was equally divided into two vials, and each was dissolved in 5 mL of alcohol—methanol, ethanol, and butanol. H_4_P_2_O_7_ (PPA) was added to one vial to prepare the acid solution (Soln1), while the other vial received the nickel salt, [Ni­(H_2_O)_6_]­(NO_3_)_2_ (NiN), to form the salt solution (Soln2). These two solutions were then mixed upon individually homogenizing to produce a clear solution containing a 1:30:15 mol ratio of P123/NiN/PPA in a total of 10 mL of alcohol. However, both the order and method of mixing (i.e., rapid one-step addition versus slow stepwise mixing) are critical to the outcome. When the two solutions are combined rapidly, the mixture initially appears clear but gradually becomes cloudy, eventually leading to a precipitation. However, when the salt solution (Soln2) is added slowly and incrementally into the acid solution (Soln1), the resulting mixture undergoes a gradual transformation from a clear solution into a gel-like phase over time—indicating a gelation.

The extent to which a clear solution is maintained varies depending on the solvent. Nonetheless, in all three alcohols—methanol, ethanol, and butanol—precipitation or gelation eventually occurs. Interestingly, when water is used as the solvent instead of alcohol, the mixture remains clear indefinitely, forming a stable solution.^31–33^ In the methanol-based system, only a small amount of precipitation was observed, regardless of the mixing order or stirring conditions. In contrast, the ethanol and butanol systems were highly sensitive to the mixing sequence and stirring method. In both ethanol and butanol media, sol–gel transitions were observed: initially clear solutions gradually transformed into gels within 1 to 15 min.

Three types of samples were collected from each solvent system to investigate the role of the solvent: (i) the whole solution (without separating the precipitate), (ii) the gel-like precipitate (isolated by centrifugation), and (iii) the supernatant (the clear liquid remaining after precipitate separation). These were analyzed to elucidate the influence of the solvent on the phase behavior and material formation (see Scheme 1). Each sample type (precipitate, whole solution, gel, and supernatant) was coated onto various substrates and analyzed throughout the alcohol evaporation process using a range of techniques, including POM, X-ray diffraction (XRD), and ATR-FTIR spectroscopy.

Figure S1 displays images of three representative samples obtained directly from the butanol system: the precipitate, the supernatant, and the whole solution. The sample from the solution phase yielded a crack-free rough coating. In contrast, the supernatant formed a smooth film upon butanol evaporation. The precipitate produced a smooth but particle-rich coating with visible cracks after 1 day of aging. This indicates that the reaction between Ni­(II) and PPA continues during aging or solvent evaporation, eventually forming a solid or gel-like phase.

Additionally, the POM images appear dark under crossed polarizers, suggesting the formation of either a cubic phase (isotropic) or a disordered phase. To further elucidate the structural details of these samples, they were also characterized by using small-angle XRD to monitor the structural evolution during gelation and aging. It is worth noting that similar behaviors were observed in all solvent systems.

Figure S2 shows the small-angle XRD patterns of the three sample types from the butanol system, recorded after 1 day of aging. The diffraction patterns show that the gel, obtained from the supernatant and coated as a very thin film onto a microscope slide, exhibits an ordered mesophase or mesostructured semisolid. In freshly coated samples, two distinct diffraction lines appear at 1.09° and 1.17°, 2θ. Within 10 min, the pattern evolves to show two new lines at 1.69° and 2.54°, 2θ, corresponding to *d*-spacings of 52.2 Å and 34.8 Å, respectively. These reflections are indexed to the (200) and (300) planes of a cubic phase, considering the POM image in Figure S1b, where the image is dark between cross-polarized light. After 1 day of aging, only the diffraction line at 1.69°, 2θ, remains, indicating a change in phase or a loss of order due to further polymerization of nickel and pyrophosphate species as butanol continues to evaporate. The extent of gelation and precipitation determines the Ni­(II) concentration in the supernatant phase. In the case of methanol, the Ni­(II) concentration remains high, resulting in distinct small-angle diffraction lines upon coating and aging. In contrast, butanol and ethanol lead to much lower Ni­(II) concentrations in the supernatant, and the corresponding gels formed are disordered, as evidenced by the lack of clear diffraction features (see Figures S2–S4).

In contrast, the XRD patterns of the samples coated from the solution and precipitate fractions of butanol do not exhibit any distinct small-angle lines, indicating a lack of long-range order. However, these samples still possess mesostructured features, as confirmed by nitrogen adsorption–desorption measurements of the corresponding calcined samples (see later discussion).

To shed light on the aging process and the nature of intermediate phases, samples were further analyzed using ATR-FTIR by depositing drops directly onto the ATR crystal. Figure 1a shows the ATR-FTIR spectra of the supernatant, solution, and precipitate samples after 24 h of butanol evaporation. Spectra in Figure 1b,c compare the ethanol and butanol solutions and supernatants, respectively, after complete solvent evaporation. All samples exhibit surfactant-related peaks, indicating the self-assembly of the polymerizing Ni_2_H_*x*_P_2_O_7_(NO_3_)_*x*_·nH_2_O particles and the P123 surfactant molecules. Absorption bands in the range of 1250–1500 cm^–1^ are attributed to nitrate species and serve as markers to monitor both the polymerization reaction between Ni^2+^ and PPA and the extent of the reaction described in eq 1.

1 $$2Ni{\left({NO}_{3}\right)}_{2}\cdot 6H_{2}O\left(sol\right)+H_{4}P_{2}O_{7}\left(sol\right)\to {Ni}_{2}H_{x}P_{2}O_{7}{\left({NO}_{3}\right)}_{x}\cdot {nH}_{2}O\left(s\right)+{HNO}_{3}\left(g\right)$$

**Figure 1:**
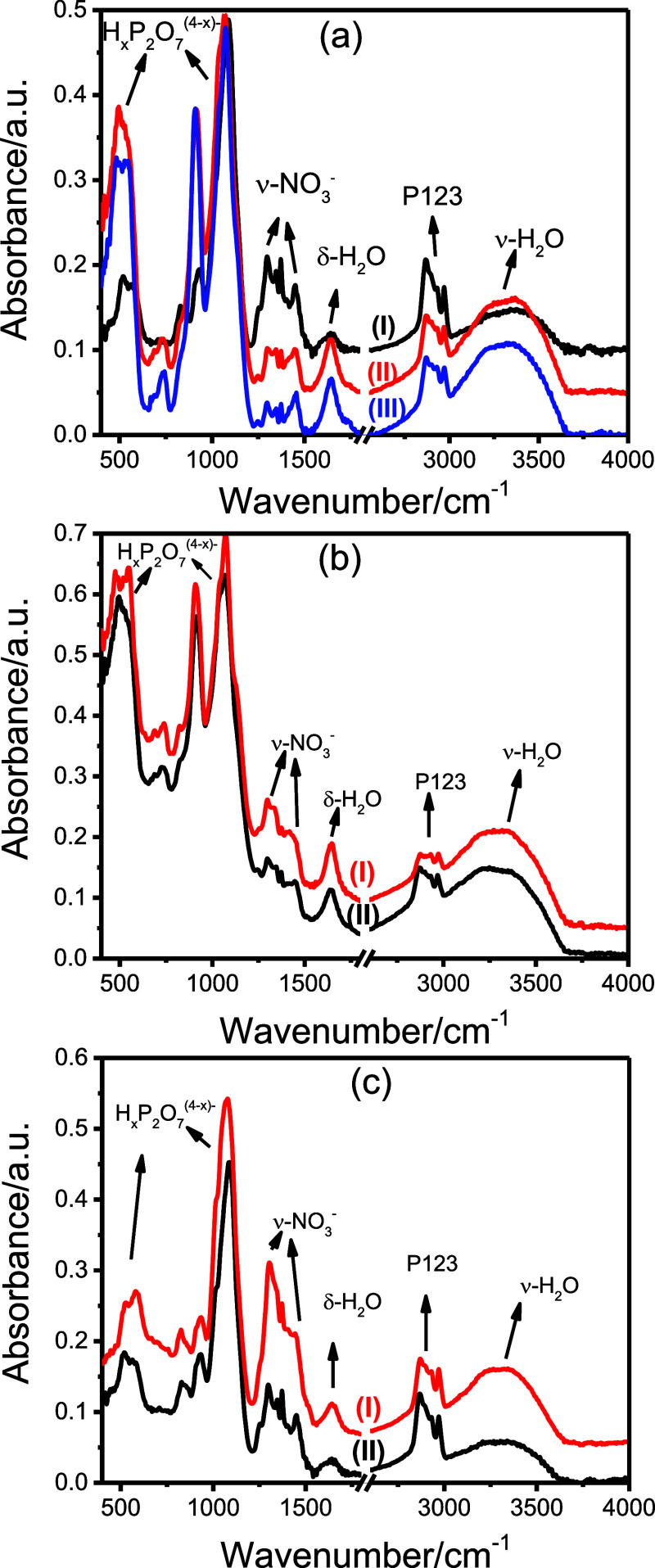
ATR-FTIR spectra of (a) (I) supernatant, (II) solution, and (III) precipitate from 1-butanol solution and (b) solutions and (c) supernatants of the (I) ethanol and (II) 1-butanol solutions.

The reaction between Ni^2+^ ions and PPA species—namely, H_3_P_2_O_7_^–^, H_2_P_2_O_7_^2–^, HP_2_O_7_^3–^, and P_2_O_7_^4–^—produces a semisolid composed of ultrasmall Ni_2_H_*x*_P_2_O_7_(NO_3_)_*x*_·nH_2_O nanoparticles associated with the P123 surfactant. This reaction also releases volatile nitric acid into the medium. As a result, the nitrate ions are removed from the media as nitric acid and its volatile decomposition products, gradually disappearing from the reaction mixture. Therefore, the nitrate peak intensity can be used as a measure of the polymerization.

The extent of this reaction is greatest in the precipitate fraction and is more pronounced in the butanol-based system as expected because the butanol system is least stable and undergoes quick gelation. The ATR-FTIR spectra show peaks in the 700–1200 cm^–1^ region corresponding to pyrophosphate species (from both PPA species and Ni_2_H_*x*_P_2_O_7_(NO_3_)_*x*_·*n*H_2_O). Therefore, the ratio of nitrate peaks (1250–1500 cm^–1^) to pyrophosphate peaks provides insight into the progress of the reaction described in eq 1. As expected, this nitrate-to-pyrophosphate ratio is highest in the supernatant and lowest in the precipitate, correlating well with the XRD observations and solution behaviors. The supernatant contains a relatively low (salt + acid)/P123 ratio and forms an ordered mesostructure.^23,24^ In contrast, the precipitate and whole-solution samples lose their solvent more quickly and form disordered mesostructures (discussed later). Notably, the precipitate contains a high amount of P123, as confirmed by ATR-FTIR spectra, supporting the idea that the surfactant molecules assist in assembling Ni_2_H_*x*_P_2_O_7_(NO_3_)_*x*_·nH_2_O nanoparticles into mesostructures during gelation and/or precipitation.

The order of mixing the clear solutions (Soln1 and Soln2) plays a crucial role in the assembly process. When the salt solution (Soln2) is slowly added to the acid solution (Soln1), rapid gelation occurs and fills the entire container within a short time, especially if gently agitated. This leads to the formation of mesostructured particles through a sol–gel process, in which the large volume of alcohol (10 mL) is retained among the mesostructured network, facilitating gelation. The structural details of the gel phase are schematically illustrated in Figure 2. Briefly, the process begins with the formation of Ni_2_H_*x*_P_2_O_7_(NO_3_)_*x*_·*n*H_2_O nanoparticles in the solution phase, which subsequently assemble with the P123 species to form larger sol particles. These sol particles then aggregate in the medium, ultimately leading to the formation of the gel phase.

**Figure 2:**
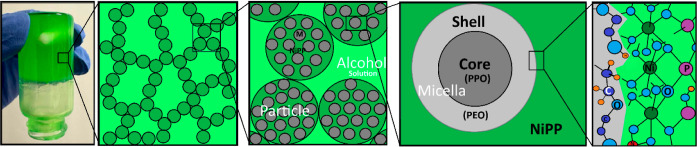
Photograph of a gel sample and schematic representations of the sample in various length scales (macroscopic to microscopic). Left to right marked square in expanded (scales are 20 mL vial, ∼5 × 5 μm^2^, ∼1 × 1 μm^2^, ∼7 × 7 nm^2^, and ∼1 × 2 nm^2^, respectively): aggregates of the spherical particles (gel-phase), a closer look at individual mesostructured particles and the surfactant-primary nanoparticle interface (shell-ethylene oxides, core-propylene oxides, and NiPP–Ni_2_H_*x*_P_2_O_7_(NO_3_)_*x*_·*n*H_2_O particles).

Conversely, reversing the order—adding the acid solution to the salt solution—delays gelation and maintains a clear solution for a longer period. However, even this configuration eventually results in precipitation or gelation (Figure S2). This tunable behavior is highly relevant for controlling the next steps of the synthesis. Understanding these fundamental processes is critical for scalable production of mesoporous Ni_2_P_2_O_7_ materials in both powder and thin-film forms. Rapid gelation can be utilized to prepare large quantities of mesoporous Ni_2_P_2_O_7_ powder, while stable solutions are ideal for coating substrates and for forming thin-film electrodes.

The coated and drop cast samples were calcined at various temperatures and characterized using XRD, ATR-FTIR, N_2_ adsorption–desorption, XPS, SEM, and transmission electron microscopy (TEM) techniques. The sol–gel solution was poured—without agitation—into a Pyrex Petri dish and dried at 100 °C for 24 h and then ground in a mortar and calcined at temperatures ranging from 300 to 700 °C. Figure 3a displays the ATR-FTIR spectra of the samples calcined at five different temperatures. The spectra display broad absorption bands characteristic of pyrophosphate units. These include stretching vibrations in the 750–1200 cm^–1^ region, symmetric and asymmetric stretching of bridging P–O–P bonds (750–950 cm^–1^) and PO_3_ units (above 950 cm^–1^), and bending modes in the 400–650 cm^–1^ range, all of which persist up to 600 °C. At 700 °C and above, these bands become sharper and split, indicating the onset of crystallization.

**Figure 3:**
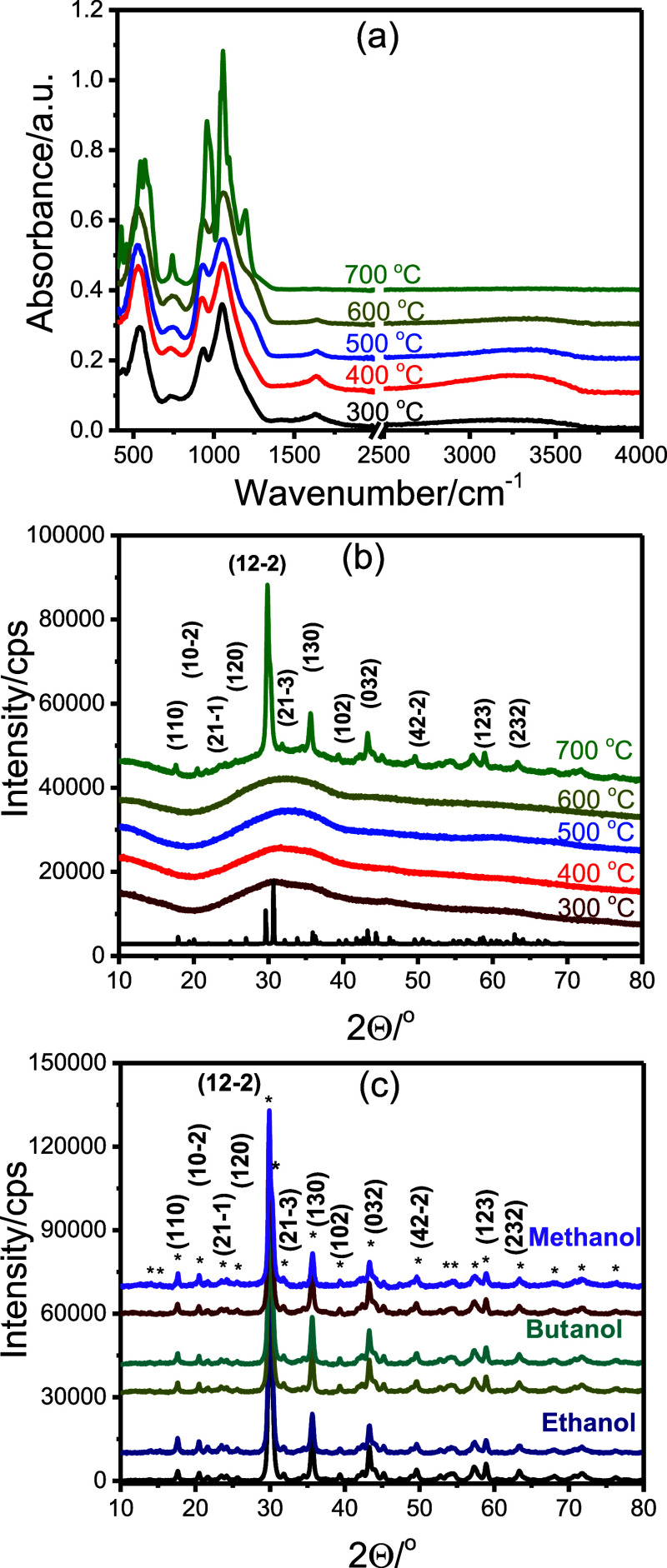
(a) ATR-FTIR spectra and (b) wide-angle XRD patterns of the mesoporous Ni_2_P_2_O_7_ at different calcination temperatures (1-butanol) and (c) calcined at 700 °C from three alcohols (methanol, 1-butanol, and ethanol, bottoms obtained from solution and tops from precipitate).

Figure 3b,c presents the wide-angle XRD patterns of the same samples along with powders synthesized from ethanol and methanol solutions. The materials remain amorphous up to 600 °C. However, above 700 °C, all samples exhibit diffraction lines corresponding to the pure α-phase of Ni_2_P_2_O_7_.^40^ These patterns are consistent across all solvent systems and can be indexed to monoclinic α-Ni_2_P_2_O_7_ (space group *P*2_1_/*c*), which is in agreement with the ATR-FTIR results. Although the mixing order and solvent type affect the porosity and textural properties (as discussed later), they do not significantly influence the microscopic structure or vibrational spectra.

The calcined powders (at 300, 400, 500, 600, and 700 °C) were further analyzed via nitrogen adsorption–desorption measurements to examine the effects of solvent and mixing sequence. Figure 4a,b displays the isotherms and pore size distribution plots for samples prepared using precipitate and solution portions of ethanol and butanol, respectively. All samples exhibit Type IV isotherms, characteristic of mesoporous materials.^41^ Textural properties such as surface area, pore-width, and pore volume vary depending on how the samples were collected. Table S1 compares all samples and literature values on surface area, pore width, and pore volume. The highest surface area was observed in samples that underwent rapid gelation. Materials formed via the sol–gel process exhibited surface areas at least twice as high and showed narrower, more uniform pore size distributions (Figure 4c and Table S1). In contrast, both precipitate and supernatant samples also formed mesoporous Ni_2_P_2_O_7_ but with smaller surface areas and broader pore distributions. Between these, the precipitates yielded average pore diameters smaller than those of the solution-derived samples (Figure 4d). It is also important to note that with increasing calcination temperature, the pores expand, become less uniform, and ultimately collapse or disappear above 700 °C, as shown in Figure 4a,b.

**Figure 4:**
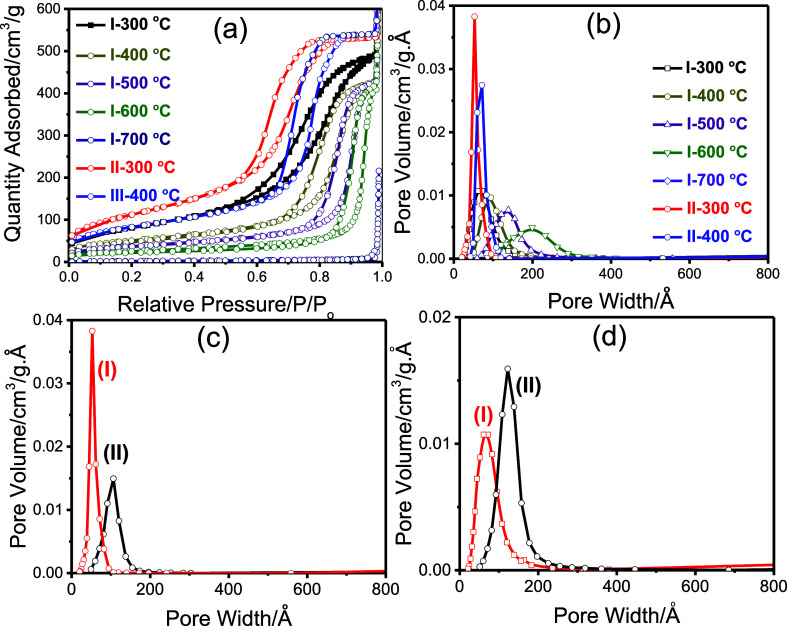
N_2_(77 K) adsorption–desorption isotherms at different calcination temperatures of the samples, obtained from (a) the sol–gel process in ethanol precipitate (I) and 1-butanol solution (II) and (b) their BJH pore size distribution plots. Comparison of the (c) BJH pore-size distribution plots of the sol–gel (I) and precipitate (II), (from 1-butanol system) and (d) compares the BJH pore-size distribution plots of the solutions of (I) ethanol and (II) methanol systems, calcined at 300 °C.

The samples were also analyzed using microscopy techniques. Figures 5 and S5 present a series of SEM images of the samples obtained by calcining the gel-phase at 300 °C and subsequently annealing at 400, 500, 600, and 700 °C. During the calcination of the gel, large monolithic particles are formed. The SEM images reveal a particle-like morphology up to 500 °C. At higher temperatures, pores begin to appear and become clearly visible (resolved) in the SEM image. However, these pores are not resolved in the N_2_ adsorption–desorption isotherms due to their large, irregular pore dimensions and the inherent limitations of N_2_ physisorption in accurately detecting pores of this size range.

Although the adsorption–desorption measurements confirm that samples are porous below 500 °C, the pores are too small to be resolved by SEM (see Figure S5). However, as the annealing temperature increases, pore expansion occurs, making the porosity visible in the images. Interestingly, despite the N_2_ isotherms showing no significant porosity in the sample annealed at 700 °C, the corresponding SEM image displays large but uniformly distributed pores throughout the particles (Figure 5). This same trend is observed in all sample types—precipitate, solution, and supernatant—across all solvents studied (Figure S6). TEM image further confirms the formation of mesoporous structures, revealing a porous architecture composed of 30–50 nm mesoporous particles aggregated into monolithic bodies and/or thin films with features on the nanometer scale (Figure 6). However, the samples are highly beam-sensitive, making it difficult to obtain high-resolution images that clearly display uniform pores, as instead evidenced by the N_2_ adsorption–desorption isotherms.

**Figure 5:**
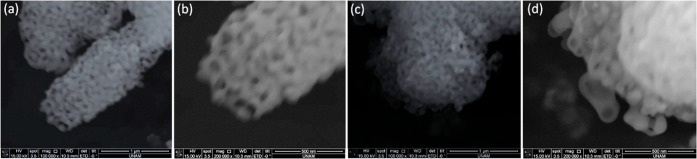
SEM images of Ni_2_P_2_O_7_ calcined at different temperatures: (a) 600 °C (scale bar is 1 μm), (b) 600 °C (scale bar is 500 nm), (c) 700 °C (scale bar is 1 μm), and (d) 700 °C (scale bar is 500 nm).

**Figure 6:**
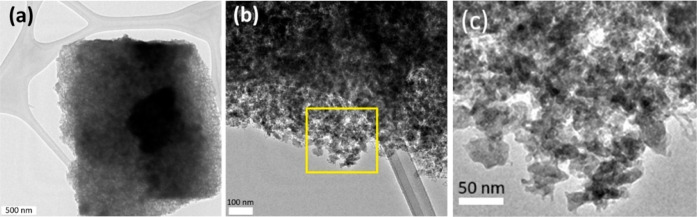
TEM image of the sample (300 °C), obtained from methanol solution, in two different magnification (a) scale bar is 500 nm, (b) scale bar is 100 nm, and (c) magnified yellow square in panel (b).

High-resolution Ni 2p, O 1s, and P 2p XPS spectra were recorded, and a representative set is shown in Figure S7. The main peak in the Ni ^2^P_3/2_ region is characteristic of the Ni^2+^ species. Likewise, the P 2p and O 1s spectra are consistent with those of Ni_2_P_2_O_7_ and exhibit a slight shift to lower binding energies, indicating the removal of hydrogens of the P–OH groups and the crystallization of the amorphous Ni_2_P_2_O_7_. These spectra further confirm that the surface composition closely matches the bulk composition of mesoporous Ni_2_P_2_O_7_.

The 1-butanol-based solution was used to fabricate electrodes by coating onto FTO glass via spin coating and onto graphite rods via dip-coating (after 5-fold dilution of the stock solution) and then calcined at various temperatures to produce FTO- and graphite-supported electrodes. The electrodes are used to investigate their electrochemical properties in 1 M KOH solution (see Figure 7a,b). However, the electrodes coated on FTO exhibit limited stability in alkaline media. The current density associated with both the redox peaks (around 0.5 V vs NHE) and OER potentials (above 0.7 V vs NHE) gradually decreases with repeated cycling. This degradation is especially pronounced for the electrode calcined at 300 °C, whereas the electrode annealed at 600 °C shows a significantly improved stability.

2 $${Ni}_{2}P_{2}O_{7}\left(s\right)+4{OH}^{-}\left(aq\right)\to 2Ni{\left(OH\right)}_{2}\left(s\right)+P_{2}O_{7}^{4-}\left(aq\right)$$

**Figure 7:**
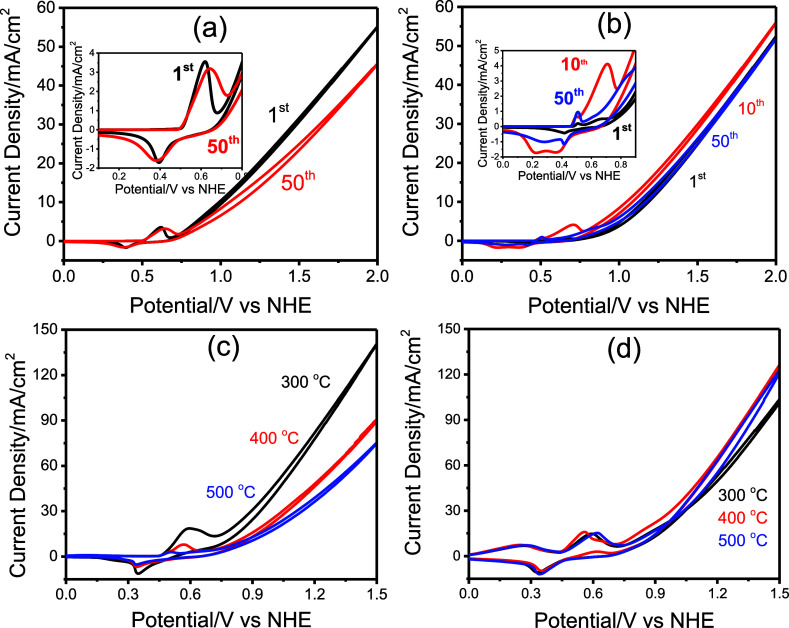
CVs of the Ni_2_P_2_O_7_ electrodes: the FTO-coated (1-butanol supernatant) electrodes, calcined at (a) 300 °C (1st and 50th CVs) and (b) 600 °C (1st, 10th, and 50th CVs, insets show the redox regions) and graphite-coated (5-fold diluted, 1-butanol solution) electrodes, calcined at 300, 400, and 500 °C (c) 1st and (d) 50th CV cycles.

As previously reported, Ni_2_P_2_O_7_ rapidly transforms into Ni­(OH)_2_ in alkaline media.^32,33^ This transformation occurs particularly quickly in electrodes calcined at 300 °C, where the peak current initially reaches its highest value (see the inset in Figure 7a). The transformation rate decreases with an increasing annealing temperature and crystallinity (compare insets in Figures 7a,b). Consequently, the peak current first increases during the transformation process (eq 2) but subsequently decreases upon further CV cycling. Despite a substantial reduction in surface area from 410 to 107 m^2^ g^–1^ at 600 °C, both electrodes (calcined at 300 and 600 °C) exhibit comparable OER performance after complete transformation (Figure 7b), suggesting the formation of similar particle sizes and morphologies in the final Ni­(OH)_2_ phase. However, determining the exact point of transformation completion is challenging due to two competing processes: an increase in current density from the Ni_2_P_2_O_7_ → Ni­(OH)_2_ transformation and a simultaneous decrease resulting from electrode degradation.

Therefore, graphite rods were also coated using a 5-fold diluted mother solution via dip-coating, calcined at 300 °C and subsequently annealed at higher temperatures. The first and 50th CVs are shown in Figure 7c,d, respectively. The first CVs reveal that the transformation (Ni_2_P_2_O_7_ to Ni­(OH)_2_) becomes progressively slower with increasing annealing temperature, which is attributed to the thickening of the pore walls: the oxidation peak currents for the electrodes calcined at 300, 400, and 500 °C are 19.7, 8.0, and 3.0 mA/cm^2^, respectively (see Figure 7c). However, after 50 CV cycles, all of the electrodes reach a similar oxidation peak current density (see Figure 7d), indicating a complete transformation in all electrodes.

A similar trend is also observed in the OER potentials (around 1.5 V (vs NHE). At 1.5 V, the current density reaches 120 mA/cm^2^ for electrodes annealed at 400 and 500 °C, which is even higher than that of the electrode calcined at 300 °C (see Figure 7d). These results suggest that despite differences in initial transformation kinetics all electrodes eventually fully transform into Ni­(OH)_2_ with comparable particle size and morphology.

The electrodes are also prepared using the supernatants. However, their electrochemical stability varies depending on the concentrations of PPA and NiN in the supernatant (see Figure 8a–d). Precipitation is more pronounced in butanol- and ethanol-based systems compared to that in methanol, resulting in greater depletion of PPA and NiN in the butanol and ethanol supernatants. In contrast, the solution and supernatant from the methanol system have similar compositions, and thus their corresponding electrodes exhibit nearly identical electrochemical behavior (compare Figure 8a,b).

**Figure 8:**
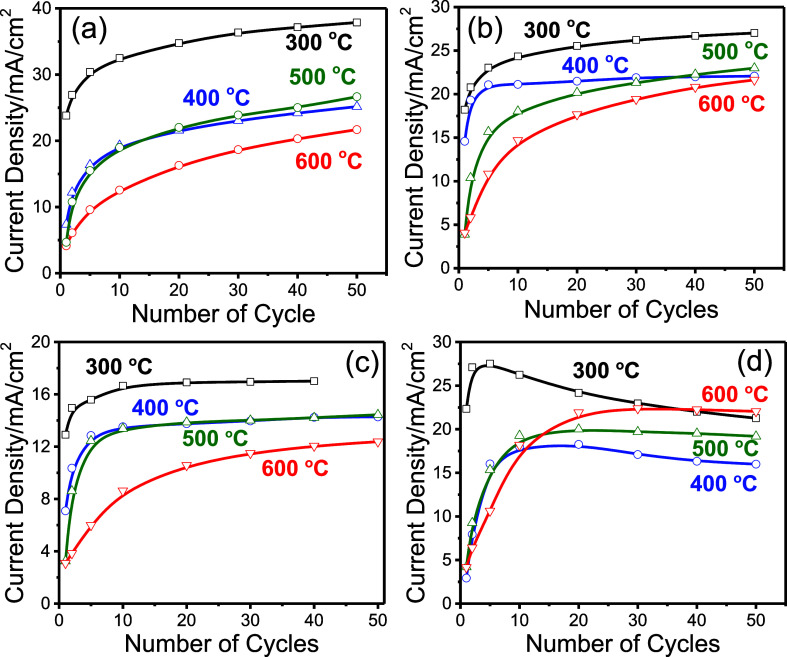
Oxidation peak current (at around 0.6 V) versus number of CV cycles of the graphite-coated Ni_2_P_2_O_7_ electrodes, prepared from methanol solution (a) and supernatant (b), and ethanol solution (c) and supernatant (d), calcined at indicated temperatures (error bars are ±1 μA).

The electrodes derived from the ethanol supernatant show a sharp increase in peak current during the first 10 cycles, followed by a gradual decline in later cycles (compare Figure 8c,d). However, annealing at 500 and 600 °C improves their stability (compare plots in Figure 8d). Overall, the electrodes, produced from the solutions or supernatants of all solvent systems (methanol, ethanol, and 1-butanol), exhibit similar long-term OER behavior. This is attributed to the complete transformation of Ni_2_P_2_O_7_ at all temperatures into Ni­(OH)_2_, resulting in electrodes with comparable Ni­(OH)_2_ particle sizes and morphologies.

The transformation of Ni_2_P_2_O_7_ into Ni­(OH)_2_ was also monitored using ATR-FTIR spectroscopy and XRD technique. For this purpose, a 0.1 g of Ni_2_P_2_O_7_ was dispersed in 10 mL of 1 M KOH aqueous solution, and samples were collected at various time intervals: immediately, 15, 30, 60 min, and after 1 day (see Figure 9). Each sample was centrifuged, washed at least four times with distilled water to remove any residual KOH, and dried at 80 °C for 1 day.

**Figure 9:**
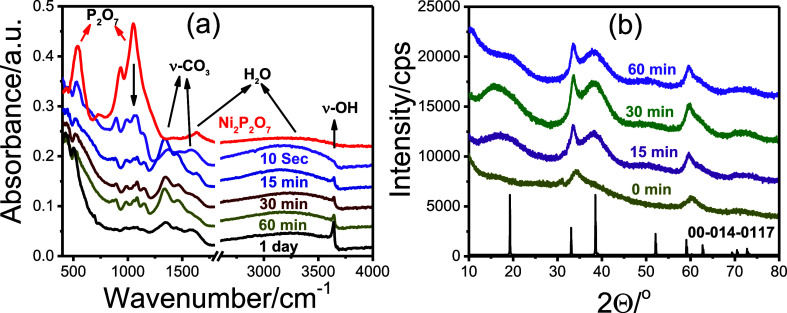
(a) ATR-FTIR spectra and (b) XRD patterns, recorded during the transformation of Ni_2_P_2_O_7_ powder in 10 mL of 1 M KOH solution over time.

Note that this experiment was conducted using a much larger amount of sample for above measurements; therefore, the transformation occurs more slowly. The time-dependent ATR-FTIR spectra (Figure 9a) show a rapid decrease in the intensity of the characteristic pyrophosphate peaks in the 800–1200 cm^–1^ and around 500 cm^–1^ regions. Simultaneously, a sharp peak at 3640 cm^–1^—attributed to isolated hydroxide (^−^OH) stretching—emerges and intensifies over time, confirming the formation of Ni­(OH)_2_. Corresponding XRD patterns (Figure 9b) reveal the appearance of broad, yet distinct, diffraction lines characteristic of β-Ni­(OH)_2_. Notably, reflections associated with the *c*-axis are very broad, while those corresponding to the *a*- and *b*-axes are relatively sharper. Also, the ATR-FTIR spectra display an intense adsorbed broad water peak with the transformation. ATR-FTIR and XRD data suggest that the transformation results in the formation of few-layer-thick β-Ni­(OH)_2_ nanoflakes.

Importantly, this transformation proceeds rapidly in a 1 M KOH solution but does not occur in a neutralized medium prepared by mixing equal volumes of 2.4 M KOH and 0.6 M H_4_P_2_O_7_ solutions (pH ≈ 11.44). To study this transformation further, a Ni_2_P_2_O_7_-coated graphite rod electrode was cycled in the neutralized solution by performing 5 consecutive CV scans. After each 5 cycles, a 0.5 mL of 1 M KOH solution was added incrementally to the electrolyte solution until complete transformation was achieved.

Figure 10 presents the fifth CVs from each step as well as a plot of the current density at 1.5 V (vs NHE) as a function of the total volume of KOH added. These results illustrate the progressive transformation of Ni_2_P_2_O_7_ into Ni­(OH)_2_ with increasing alkalinity in the electrolyte media. The current density at 1.5 V gradually increases with the incremental addition of KOH to the neutralized electrolyte solution, indicating that the OER is highly dependent on the hydroxide ion concentration. Notably, the Ni_2_P_2_O_7_ electrode is stable in the neutralized solution (pH ≈ 11.44), and also with up to approximately 3 mL of 1 M KOH addition (Figure 10a). Presence of a high concentration of P_2_O_7_^4–^ ions (about 0.6 M) in the medium suppresses this transformation. Beyond this point (pH ≈ 13.13), the CVs begin to show noticeable changes, consistent with the formation of Ni­(OH)_2_ species on the electrode surface. A new oxidation peak appears at around 1.2 V after the addition of 3 mL of KOH, and its current density continues to increase with further KOH additions up to 8.5 mL (Figure 10b,c). In the reverse scan (reduction cycle), this peak reappears at a similar potential with a smaller, yet still with a positive current density.

**Figure 10:**
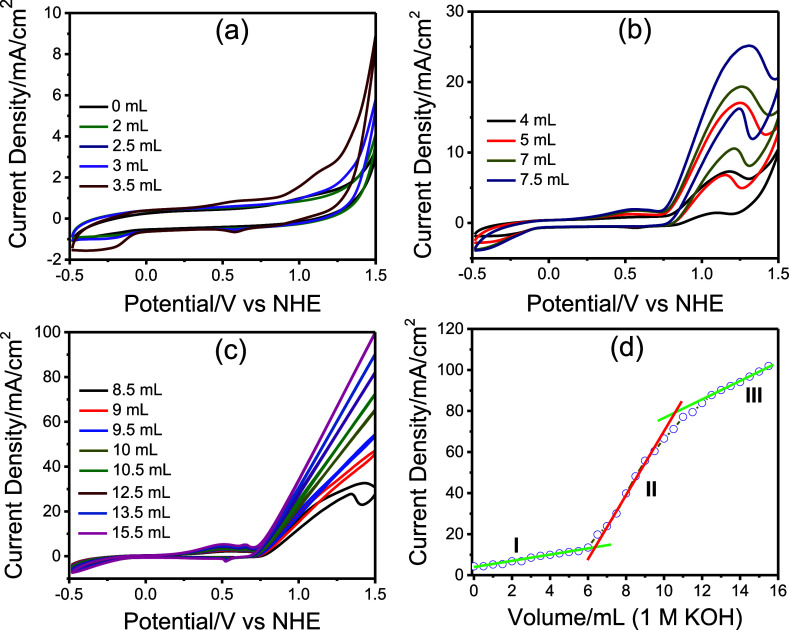
5th CVs after addition of (a) 0–3.5 mL, (b) 4–7.5 mL, and (c) 8.5–15.5 mL addition of 1 M KOH to neutralized (2.4 M KOH + 0.6 M H_4_P_2_O_7_ solution) electrolyte solution and (d) plot of current density versus added KOH at 1.5 V.

We attribute this newly observed peak at ∼1.2 V to the OER activity of the newly formed Ni­(OH)_2_ species on the electrode surface. In this region, the electrode surface consists of both Ni_2_P_2_O_7_ and Ni­(OH)_2_ species. Note that a similar behavior is observed during alcohol oxidation in an alkaline solutions,^42,43^ where the limiting factor is the alcohol concentration. In the present case, the limitation arises from the gradual formation of Ni­(OH)_2_ species during the addition of 3 to 8.5 mL of KOH solution. When the surface becomes fully covered with Ni­(OH)_2_ (after adding around 10 mL of 1 M KOH, Figure 10c), the current density increases linearly with further KOH addition in the OER region.

Overall, the current density associated with the OER over Ni_2_P_2_O_7_ electrodes increases with KOH addition, ultimately reaching levels comparable to those of pure Ni­(OH)_2_ electrodes. The plot of current density at 1.5 V vs volume of KOH added is given in Figure 10d. Three distinct regions are observed in this plot: (i) region I (0–3 mL KOH), a gradual increase in current density, attributed mainly to the increased hydroxide ion concentration in the electrolyte: Ni_2_P_2_O_7_ is stable in this region, (ii) region II (3–10 mL KOH), a steep rise in current density, due to both increasing OH^–^ ion concentration and the formation of more active Ni­(OH)_2_ sites on the electrode surface, and (iii) region III (>10 mL KOH), the slope decreases again, indicating a slower transformation of residual Ni_2_P_2_O_7_ to Ni­(OH)_2_ and with continued contributions from increasing OH^–^ ion concentration.

The reduction peak current (Ni^3+^/Ni^2+^) at 0.5 V versus the volume of the added 1 M KOH solution is also plotted (see Figures 11a and S8). The peak current begins to increase noticeably after the addition of 6 mL KOH and becomes steeper between 6- and 9 mL KOH addition. Beyond this range, the current density continues to increase but with a lower slope. The derivative of this plot peaks at around 7.5 mL of KOH, with electrolyte composition—[P_2_O_7_^4–^] = 0.24 M and [OH^–^] = 0.20 M—for the transformation of Ni_2_P_2_O_7_ to Ni­(OH)_2_. The solubility product (*K*_sp_) of Ni_2_P_2_O_7_ can be calculated using these concentrations and the known *K*_sp_ value of Ni­(OH)_2_ (5.48 × 10^–16^ M^3^). Assuming the equilibrium (Ni_2_P_2_O_7_(s) ⇌ 2 Ni^2+^(aq) + P_2_O_7_^4–^(aq)), we calculate [Ni^2+^] is equal to *K*_sp_(Ni­(OH)_2_)/[OH^–^]^2^, giving a value of 1.37 × 10^–14^ M. This yields *K*_sp_(Ni_2_P_2_O_7_) of 4.5 × 10^–29^ M^3^ ([Ni^2+^]^2^ × [P_2_O_7_^4–^] = (1.37 × 10^–14^)^2^ × 0.24) that is very close to our previously reported (3.5 × 10^–29^ M^3^) value.^32^

**Figure 11:**
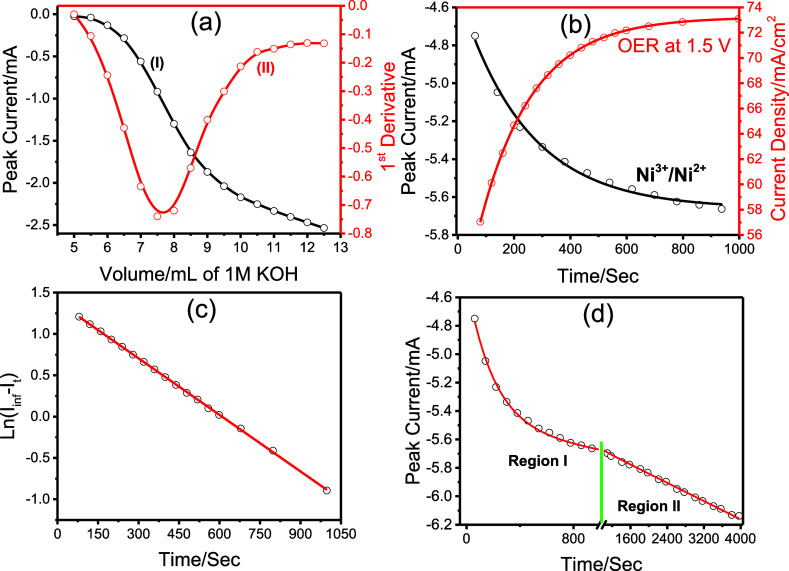
(a) Plot of peak current versus volume of KOH (collected from 5th CVs after each addition) and its 1st derivative. (b) Reduction (Ni^3+^/Ni^2+^) peak current (obtained from the CVs) versus time (black) and current density at 1.5 V versus time (red) plots (CVs were recorded in a solution of a 30 mL neutralized solution (15 mL of 0.6 M H_4_P_2_O_7_ and 15 mL of 2.4 M KOH) and additional 5 mL of 3 M KOH and (c) plot of ln­(*I*_∞_–*I*_t_) versus time obtained from the red curve in panel (b), and (d) plot of the reduction peak current versus time.

The time-dependent transformation is also carried out in a more alkaline solution by adding 5 mL of 3 M KOH to the neutralized solution, resulting in a composition of 0.26 M P_2_O_7_^4–^ and 0.43 M OH^–^. Figure 11b presents the evolution of OER current density at 1.5 V and the Ni^3+^/Ni^2+^ reduction peak current over time. The OER current increases exponentially and stabilizes after approximately 1000 s. A plot of ln­(*I*_∞–_ *I*_t_) versus time, where *I*_∞_ is the steady-state current (obtained by fitting the data to an exponential function, *I*_t_ = *I*_∞_ + *A*e^*Bt*^, where *I*_∞_ = 73.228, *A*= −24.706, *B*= −0.00528, and *t* is time in sec with an *R* ^2^ of 0.99994) and *I*_*t*_ is the current at any time *t*, shows linear behavior, indicating a first-order kinetic with a rate constant of 2.28 × 10^–3^ s^–1^ (see Figure 11c).

Interestingly, the time-dependent evolution of the Ni^3+^/Ni^2+^ reduction peak current (Figure 11d) mirrors the OER current in the early stage (Region I, up to 1000 s). However, in subsequent CV cycles (Region II), the peak current continues to increase linearly, even when the surface is fully covered with Ni­(OH)_2_. This suggests that while the surface transformation drives the OER activity, the interior transformation of Ni_2_P_2_O_7_ into Ni­(OH)_2_ continues and is captured in the growing redox peak currents. These sites are not accessible for the OER but the redox reaction (Ni­(OH)_2_(s) + OH^–^(aq) → NiOOH(s) + H_2_O­(l) + e^–^) still occurs. After surface coverage is complete, the reaction kinetics transitions to zero-order, governed by the bulk transformation. Further investigations are required to fully elucidate the mechanism and kinetics of the Ni_2_P_2_O_7_ to Ni­(OH)_2_ transformation process to establish the foundation of the high OER efficiency and charge-capacity in alkaline media.

The graphite electrode, prepared from the butanol-based solution, was further analyzed to evaluate its electrochemical behavior and OER performance. Figure 12 presents the results of multistep chronopotentiometry (m-CP) and multistep chronoamperometry (m-CA), used to determine the overpotentials and Tafel slopes, respectively. The m-CP experiments were performed at current densities of 1, 10, 20, 30, 40, 50, 60, 70, 80, 90, and 100 mA/cm^2^. Prior to these measurements, 50 CV cycles were conducted to ensure a complete transformation of the electrode. Then, 60 min CP experiments were carried out at each current density (Figure 12a). At 1 mA/cm^2^, the overpotential gradually decreased—possibly indicating further transformation or enhanced OER performance—and stabilized at around 273 mV. At higher current densities, a slight increase in overpotentials was observed. The overpotential vs current density plot follows an exponential trend; however, subtracting the Tafel slope reveals a linear relationship with a slope of 5.08 Ω, corresponding to the series resistance of the cell; see Figure 12b. A more detailed analysis shows a slight decrease in resistance to approximately 4.8 Ω at higher current densities. Correcting for IR drop using these resistance values yields an overpotential of 381 mV at 100 mA/cm^2^ and indicates an excellent OER kinetics. The Tafel plot (Figure 12c) displays a slope of 45 mV/dec, which is relatively low and also indicative of excellent OER kinetics. When the overpotential and Tafel slope are compared with those of Ni­(OH)_2_ electrodes—either prepared directly as Ni­(OH)_2_ or derived from Ni­(OH)_2_ over Ni, NiO, or Ni_1–*x*_Mn_*x*_O electrodes^44–48^ —the Ni_2_P_2_O_7_-derived Ni­(OH)_2_ exhibits higher purity and lower overpotential and Tafel slope (Table S2). Therefore, the transformation reaction described above provides a new route to synthesize highly active nickel hydroxide from various nickel compounds. Extending this concept to the metal–organic framework (MOF) family, this approach could enable novel synthesis and electrode fabrication strategies for metal hydroxides with tunable purity and morphology.

**Figure 12:**
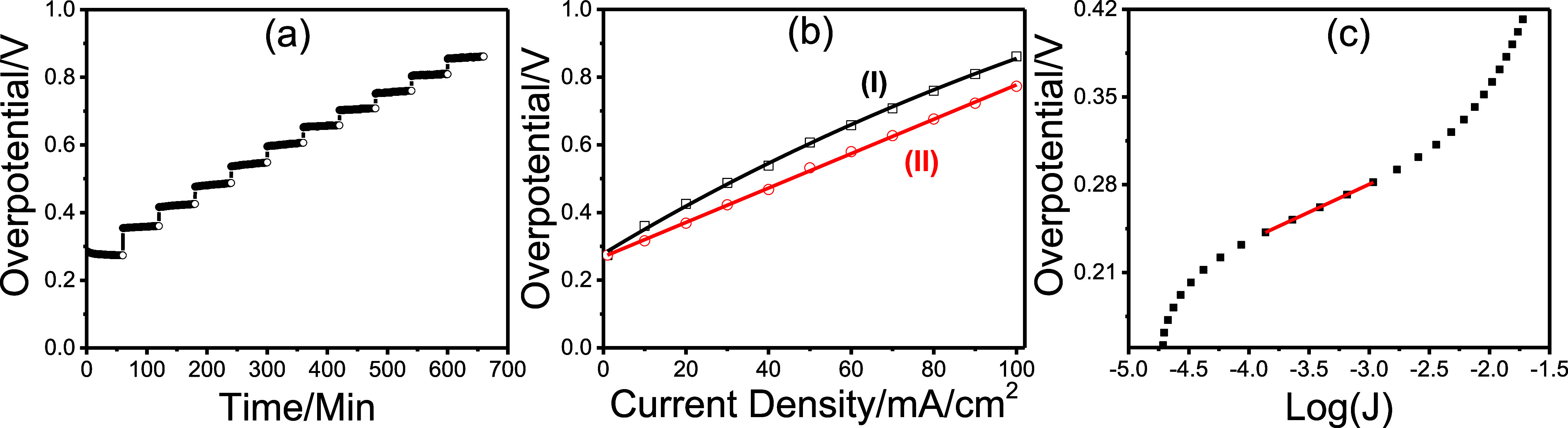
Graphite electrode obtained from the butanol system: (a) m-CP at 1, 10, 20, 30, 40, 50, 60, 70, 80, 90, and 100 mA/cm^2^ current densities, (b) plot of (I) overpotential versus current density and (II) Tafel corrected overpotential versus current density (with an error bar of ±25 mV on the *y*-axis), and (c) multistep chronoamperometry curve.

Mechanistically, Ni­(OH)_2_ is oxidized to NiOOH (eq 3), which can undergo further oxidation to form nickel–oxo (Ni=O) species on the electrode surface in an alkaline medium. These nickel–oxo sites are crucial for the OER electrocatalysis. The electrochemically generated oxo-oxygen becomes highly electrophilic (Ni=O^δ+^) with further oxidation of Ni^4+^ sites and reacts nucleophilically^49–53^ with hydroxide ions to produce a Ni–O–O–H intermediate, representing the O–O bond formation step. In this process, Ni^5+^ is reduced back to Ni^3+^, while the oxygen atoms are oxidized to form a peroxide species. The likely Ni^5+^ intermediates are tetrahedral (O_3_Ni=O), where the Ni=O bond serves as the active site, and the remaining oxygen atoms act as lattice oxygens anchoring the active species to the electrode surface.

3 $$Ni{\left(OH\right)}_{2}\left(s\right)+{OH}^{-}\left(aq\right)\to NiOOH\left(s\right)+H_{2}O\left(l\right)+e^{-},oxidation$$

4 $$NiOOH\left(s\right)+e^{-}+H_{2}O\left(\mathrm{or}H_{3}O^{+}\right)\left(aq\right)\to Ni{\left(OH\right)}_{2}\left(s\right)+{OH}^{-}\left(\mathrm{or}H_{2}O\right)\left(aq\right),reduction$$

Upon further electrochemical oxidation of the Ni–O–O–H intermediate (where Ni^3+^ is oxidized to Ni^4+^ by releasing a H^+^ ion and then to Ni^5+^), a molecular oxygen is generated and released from the electrode surface through the above redox process. This step involves oxidation of the peroxide to aqueous O_2_ and simultaneous reduction of the Ni^5+^ sites (O_3_Ni–O–O) back to Ni^3+^, which is observed as a reduction peak in the reverse cycle of the cyclic voltammogram. The major reduction peak around 0.4 V (vs NHE) corresponds to the Ni^3+^/Ni^2+^. Notably, the observed high charge capacity—approaching the theoretical value—under these redox peaks indicates a highly efficient reversible process. During oxidation, Ni­(OH)_2_ is converted to NiOOH by hydroxide ion diffusion and reaction with Ni­(OH)_2_ layer to produce a water molecule (eq 3); in the reverse process, NiOOH is reduced back to Ni­(OH)_2_ via the diffusion of H_2_O or H_3_O^+^ ions, regenerating OH^–^ or H_2_O (eq 4), respectively. This redox cycling represents a typical battery action, enabling Ni­(OH)_2_ to function as an effective charge storage material. The same reversible redox process underlies the excellent electrochromic^47^ and supercapacitive behavior^33^ of Ni­(OH)_2_. However, water oxidation occurs only at the electrode surface—not all nickel sites are catalytically active for the OER unless an accessible single or double layer Ni­(OH)_2_ is produced.

This mechanism is consistent with our previously proposed OER pathway.^51–53^ In this model, the electrocatalytic activity and corresponding overpotential are strongly influenced by the electronegativity of the Ni=O sites. The calculated electronegativity values are 6.19 eV for Ni­(OH)_2_ and 6.48 eV for Ni_2_P_2_O_7_,^54^ indicating that Ni_2_P_2_O_7_ requires a higher overpotential for its activation (oxidation of nickel sites to Ni^4+^/Ni^5+^), as also observed in the CVs. The electroactive (O_3_Ni=O) sites are bonded to the lattice Ni atoms (lattice-(NiO)_3_Ni=O) in the hydroxide system, whereas in pyrophosphate-derived samples, they are bonded to P atoms (lattice-(PO)_3_Ni=O). Since the Mulliken electronegativity of Ni (4.40 eV) is lower than that of P (5.62 eV), the Ni–O–P bonding environment makes these sites more difficult to oxidize. Therefore, nickel pyrophosphate requires higher overpotential for the OER and is not as good electrocatalyst as Ni­(OH)_2_ electrodes.

## Conclusion

A mixture of H_4_P_2_O_7_, [Ni­(H_2_O)_6_]­(NO_3_)_2_, and P123 surfactant forms a clear solution in methanol, ethanol, and butanol when the acid and salt components are prepared in separate vials. However, upon combining the solutions, the mixtures become unstable: undergoing gelation in butanol and ethanol, and precipitation in methanol. Gelation in ethanol is relatively slow but can be accelerated by reducing the ethanol volume or modifying reaction parameters (such as increasing the pH of the solution phase).

Calcination of the gel phase yields mesoporous Ni_2_P_2_O_7_ with a record-high-surface area (410 m^2^/g) and uniform pore structures. In methanol and partly ethanol systems, where precipitation occurs, the resulting phases (precipitates and supernatants) can still be used to produce mesostructured semisolid Ni_2_H_*x*_P_2_O_7_(NO_3_)_*x*_·*n*H_2_O, which upon calcination forms mesoporous powders or thin films. Samples derived from the sol–gel route possess uniform pores (fwhm = ∼1.4 nm), while those from precipitates or solution routes tend to have larger and less uniform pore (fwhm = ∼3.4 nm) structures. The Ni_2_P_2_O_7_ phase remains amorphous up to 600 °C and crystallizes into pure α-Ni_2_P_2_O_7_ above 700 °C.

Both solutions and supernatants can be coated onto various substrates to create mesoporous thin-film electrodes. The coated Ni_2_P_2_O_7_ film is stable on graphite rod and but, still undergoes in situ transformation to Ni­(OH)_2_ that has excellent OER performance. The transformation can be controlled by tuning P_2_O_7_^4–^ and OH^–^ concentrations (0.30–0.24 and ∼3.0 × 10^–4^–0.2 M, respectively) in the electrolyte. Electrode covered with Ni­(OH)_2_ demonstrates superior OER activity compared to its Ni_2_P_2_O_7_ counterparts (current density increases by 50 times at 1.5 V). This is also a new way to prepare Ni­(OH)_2_ thin film electrodes that are difficult to prepare by other methods. This work opens up a new synthetic approach for the fabrication layered 2D metal hydroxide electrodes from various metal compounds, including large number of MOFs. Further investigation is warranted to fully understand the role of the gelation process, which may be extended to other transition-metal pyrophosphates and phosphates to improve their porosity, surface area, and electrocatalytic performance.

## Supplementary Material



## Notes

Additional POM, small-angle XRD patterns, SEM, and electrochemical data (PDF)

## Abbreviations

OER: oxygen evolution reaction
CP: chronopotentiometry
CA: chronoamperometry
BET: Brunauer–Emmett–Teller
BJH: Barrett–Joyner–Halenda
