# Supplementary material for: Scalable Solvent-Mediated Nanoarchitectonics of High-Surface-Area Mesoporous Ni2P2O7 for Enhanced Electrochemical Performance in Alkaline Media
Source: Inorg Chem. 2025 Dec 26;65(1):938–51. doi: 10.1021/acs.inorgchem.5c05373 (PMC12801325; doi:10.1021/acs.inorgchem.5c05373)
Supplement: Supplementary file 1 [file ic5c05373_si_001.pdf]

Supporting Information

# Scalable Solvent-Mediated Nanoarchitectonics of High–Surface-Area Mesoporous $Ni_2P_2O_7$ for Enhanced Electrochemical Performance in Alkaline Media

*Gözde Ceran[a], Irmak Karakaya Durukan[a], Işıl Ulu[a], Ömer Dag*[a,b]*

[a]Department of Chemistry, Bilkent University, 06800, Ankara, Turkey

[b]UNAM — National Nanotechnology Research Center and Institute of Materials Science and Nanotechnology, Bilkent University, 06800, Ankara, Turkey
Email: dag@fen.bilkent.edu.tr

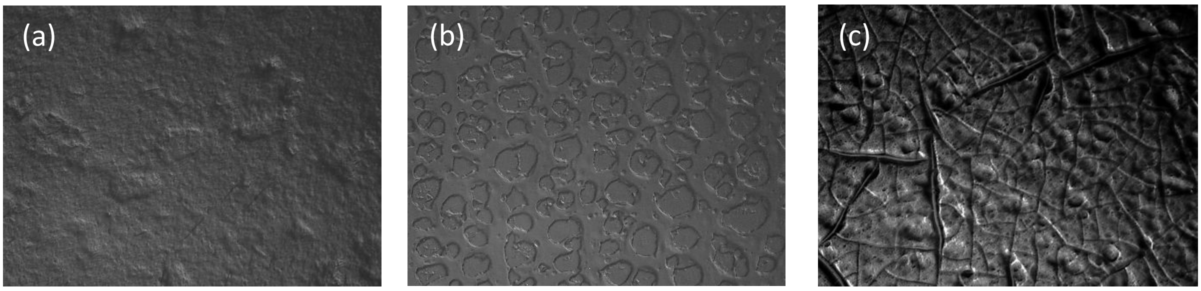


**Figure S1.** POM images of the Ni(II):PPA:P123 (1:30:15) sample in the butanol: (a) solution, (b) supernatant, and (c) precipitate forms after 1 day aging (recorded using 10x magnification).

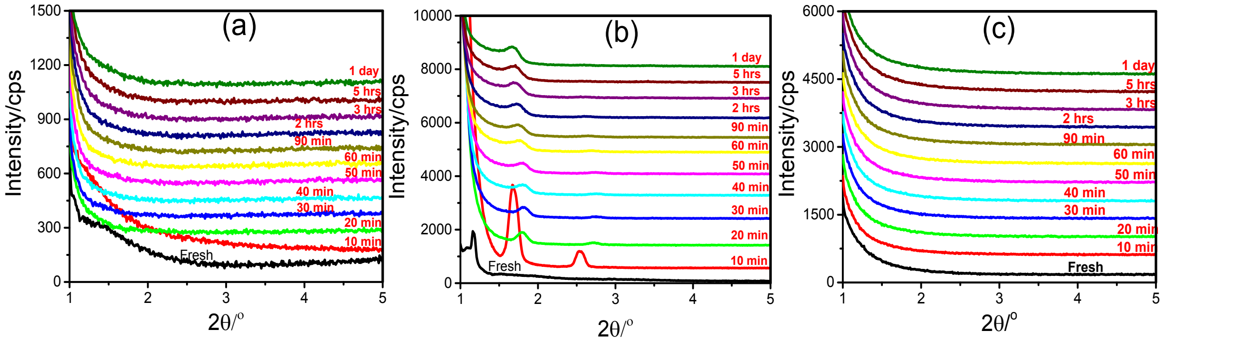


**Figure S2.** Time-dependent small angle XRD patterns of the Ni(II):PPA:P123 (1:30:15) samples from the butanol (a) solution, (b) supernatant, and (c) precipitate parts.

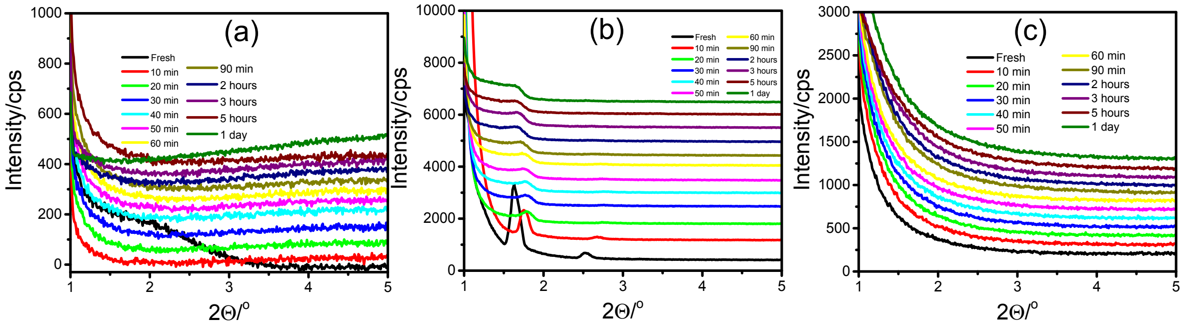


**Figure S3.** Time-dependent small angle XRD patterns of the Ni(II):PPA:P123 (1:30:15) samples from the ethanol (a) solution, (b) supernatant, and (c) precipitate parts.

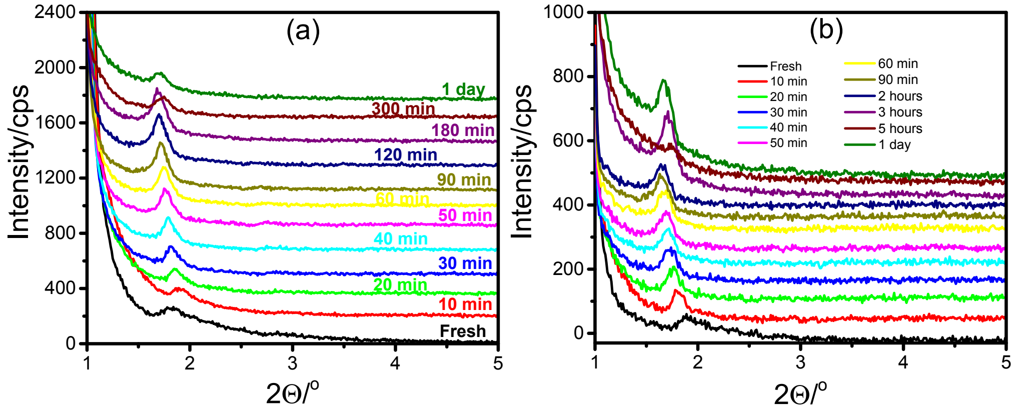


**Figure S4.** Time-dependent small angle XRD patterns of the Ni(II):PPA:P123 (1:30:15) samples from the methanol (a) solution and (b) supernatant parts.

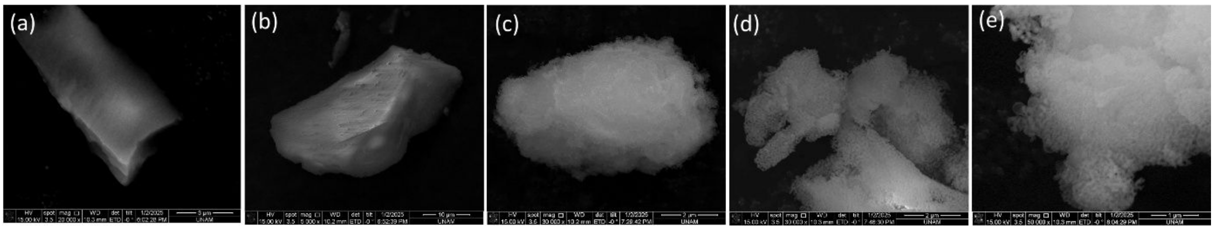


**Figure S5.** SEM images of $Ni_2P_2O_7$ calcined at different temperatures: (a) 300 °C (scale bar is 3 μm), b) 400 °C (scale bar is 10 μm), c) 500 °C (scale bar is 2 μm), d) 600 °C (scale bar is 2 μm), and e) 700 °C (scale bar is 1 μm).

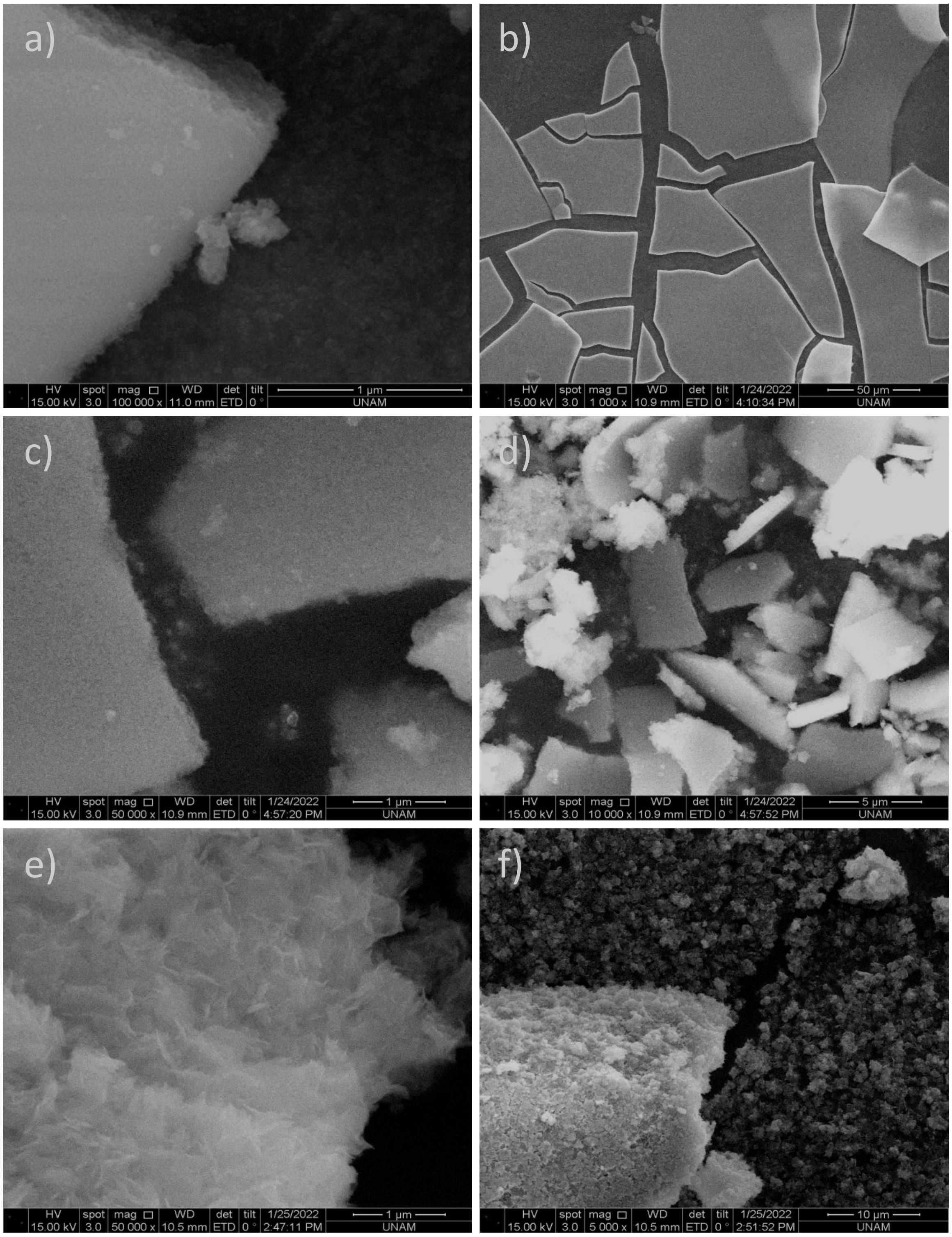


**Figure S6.** SEM images of the $Ni_2P_2O_7$ samples, calcined at 300 $^oC$, obtained from ethanol and methanol (a and b) supernatant, (c and d) solution and (e and f) precipitate.

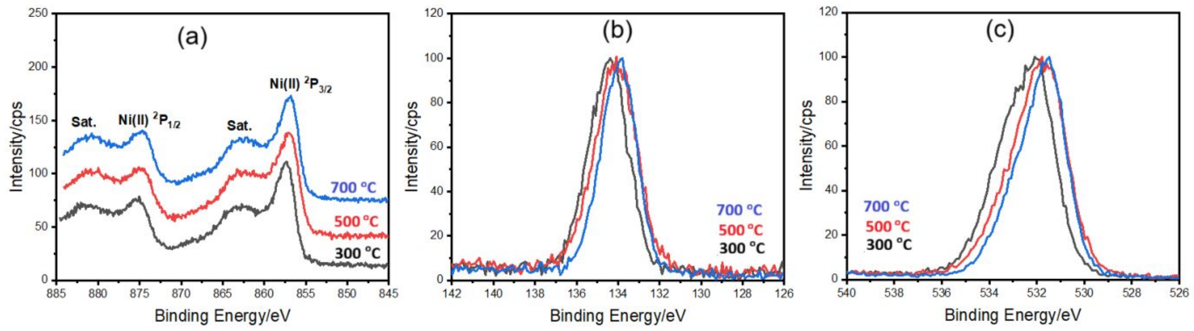


**Figure S7.** XPS spectra of $Ni_2P_2O_7$, calcined at 300, 500, and 700 °C (a) Ni 2p, (b) P 2p, and (c) O 1s regions.

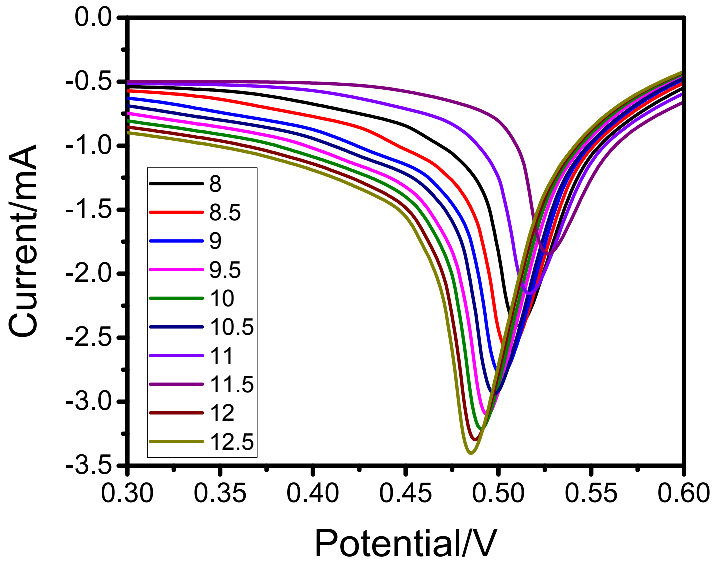


**Figure S8.** 5th CVs in the $Ni^{3+}/Ni^{2+}$ reduction peaks after incremental addition of 1M KOH solution to the neutralized (equal volume of 2.4M KOH and 0.6M $H_4P_2O_7$) electrolyte solution.

**Table S1.** BET Surface Area, BJH Pore Width, and Pore Volume Data of $Ni_2P_2O_7$. *Calcined at 300/400/500/600/700 $^oC$. G-gel, P-precipitate.

| **Sample** | **BET surface Area m$^2$/g** | **Pore Width/nm** | **Pore Volume/cm$^3$/g** | **Reference** |
|---|---|---|---|---|
| $Ni_2P_2O_7$ | 36 | - | - | 1 |
| Na-doped $Ni_2P_2O_7$ | 190 | 2-4 | - | 2 |
| $Ni_2P_2O_7$ | 30 | - | - | 3 |
| $Ni_2P_2O_7$ | 6 | 11 | | 4 |
| $Ni_2P_2O_7$ | 97/45/17 | - | 0.087/0.113/0.179 | 5 |
| $Ni_2P_2O_7$-LLC | 60/35/38 | 10.8/9.3 | - | 6 |
| $Ni_2P_2O_7$-LLC | 31/25/5 | | - | 7 |
| $Ni_2P_2O_7$ | 95 | 2 | - | 8 |
| $Ni_2P_2O_7$/RGO | 176 | 8 | - | 8 |
| $Ni_2P_2O_7$-Butanol-G* | 410/299/184/107/18 | 5.3/7.3/10.6/17.4 | 1.15/1.35/1.28/1.21/0.42 | This Work |
| $Ni_2P_2O_7$-Ethanol-P* | 297/184/134/91/8 | 6.8/9.4/13.8/20/- | 1.26/1.11/1.06/1.10/0.94 | This Work |
| $Ni_2P_2O_7$-Metahnol-P* | 240/147/108/73/20 | 10.6/11.8/14.6/23/- | 1.09/1.06/1.1/1.05/- | This Work |

**Table S2.** Overpotential and Tafel Slope comparison.

| Electrode Material/Substrate | j (mA/cm$^2$) | KOH Conc. (M) | Overpotential (mV) | Tafel Slope (mV/dec) | Reference |
|---|---|---|---|---|---|
| $Ni(OH)_2$-MD/Carbon Paper | 10 | 1 | 300 | 104 | 9 |
| $\beta$-$Ni(OH)_2$/Carbon Paper | 10 | 1 | 380 | 153 | 9 |
| NiFe-SW/Glassy Carbon | 12 | 1 | 250 | 38.9 | 10 |
| NiFe Film/Nickel Foam | 24 | 1 | 250 | - | 11 |
| a-$Ni(OH)_2$/Glassy Carbon | 10 | 0.1 | 331 | - | 12 |
| Ni-Fe Oxide/Carbon Paper | 10 | 1 | 303 | 58.5 | 13 |
| $Ni_{1-x}Mn_xO$/Graphite | 10 | 1 | 311 | 48 | 14 |
| $Ni(OH)_2$-NiPP/Graphite | 100 | 1 | 381 | 45 | This Work |

References


1) S.J. Marje, P.K. Katkar, S.S. Pujari, S.A. Khalate, A.C. Lokhande, U.M. Patil, Regulated micro-leaf like nickel pyrophosphate as a cathode electrode for asymmetric supercapacitor, *Synth. Met.* **2020**, *259*, 116224.
2) Wei, C.; Cheng, C.; Wang, S.; Xu, Y.; Wang, J.; Pang, H. Sodium-Doped Mesoporous $Ni_2P_2O_7$ Hexagonal Tablets for HighPerformance Flexible All-Solid-State Hybrid Supercapacitors. *Chem. Asian J.* **2015**, *10*, 1731.
3) C. Wei, S. Yang, W. Liu, X. Hou, Y. Sun, J. Zhao, W. Xiong, C. Cheng, D. Zhang, Hierarchically porous bowknot-like sodium doped $Ni_2P_2O_7$ - $Co_2P_2O_7$ with improved supercapacitor performances, *Appl. Surf. Sci.* **2019**, *465*, 763.
4) Senthilkumar, B.; Khan, Z.; Park, S.; Kim, K.; Ko, H.; Kim, Y. Highly Porous Graphitic Carbon and $Ni_2P_2O_7$ for a High-Performance Aqueous Hybrid Supercapacitor. *J. Mater. Chem. A* **2015**, *3*, 21553.
5) Nivetha, S.; Prabahar, S.; Karunakaran, R. T.; Ganth, M. N.; Dhinesh, S. Synthesis and Characterization of $Ni_2P_2O_7$ Thin Film as a Superier Electrode Materials for high Performance Supercapacitors. *Ionics* **2023**, *29*, 1209.
6) Ulu, I.; Ulgut, B.; Dag, Ö. Nanoarchitectonics of Mesoporous $M_2P_2O_7$ (M = Mn(II), Co(II), and Ni(II)) and $M_{2-x}Co_xP_2O_7$ and Transformation to Their Metal Hydroxides with Decent Charge Capacity in Alkali Media. *Inorg. Chem.* **2023**, *62*, 16994.
7) Ulu, I.; Ulgut, B.; Dag, Ö. Fabrication of Mesoporous Nickel Pyrophosphate Electrodes and Their Transformation to Nickel Hydroxide with Decent Capacitance in Alkaline Media. *J. Mater. Chem. A* **2023**, *11*, 22384.

8) Nair, M. S. S.; Sivakumar, T.; Venkateshwari, P. Regulated Micro-balls Like Nickel Pyrophosphate/reduced Graphene Oxide ($Ni_2P_2O_7$@RGO) Composite Thin Films as a Cathode Electrode for Asymmetric Supercapacitor. *J. Mater. Sci: Mater. Electron.* **2025**, *36*, 1055.
9) Jena, R. *et.al.* In Situ Tracking of Ni-MOF Reconstruction into Active $Ni(OH)_2$ OER Catalysts**.** *Angew. Chem. Int. Ed.* **2025,** *64*, e202510741**.**
10) Zhang, W. et al. A Thin NiFe Hydroxide Film Formed by Stepwise Electrodeposition Strategy with Significantly Improved Catalytic Water Oxidation Efficiency. *Adv. Energy Mater.* **2017**, *7*, 160254.
11) Lu, X.; Zhao, C. Electrodeposition of Hierarchically Structured Three-dimensional Nickel–iron Electrodes for Efficient Oxygen Evolution at High Current Densities. *Nature Commun.* **2015**, *6*, 6616.
12) Gao, M. *et al.* Efficient water oxidation using nanostructured α-nickel-hydroxide as an electrocatalyst. *J. Am. Chem. Soc.* **2014**, *136*, 7077.
13) Kang, B. K. *et al.* Mesoporous Ni-Fe oxide multi-composite hollow nanocages for efficient electrocatalytic water oxidation reactions. *J. Mater. Chemistry A* **2017**, *5*, 4320.
14) Katırcı, A. A.; Durukan, I. K.: Dag, Ö. Nanoarchitectonic Mesoporous $Ni_{1-x}Mn_xO$ Electrodes: Charge Capacity and Oxygen Evolution Reaction Electrocatalysis in Alkaline Media. *ACS Appl. Energy Mater.* **2025**, *8*, 3162.
